# Evaluation of one-point fixation for zygomaticomaxillary complex fractures using a three-dimensional photogrammetric analysis

**DOI:** 10.1186/s40463-019-0359-2

**Published:** 2019-07-30

**Authors:** Se Young Kim, Seung Min Nam, Eun Soo Park, Yong Bae Kim

**Affiliations:** Department of Plastic and Reconstructive Surgery, Soonchunhyang University Bucheon Hospital, 170 Jomaru-ro, Bucheon, 14584 Republic of Korea

**Keywords:** Zygomaticomaxillary complex fracture, Zygomaticomaxillary buttress, One-point fixation, Three-dimensional photogrammetric analysis

## Abstract

**Background:**

The goal of treatment for zygomaticomaxillary (ZM) complex (ZMC) fractures is to achieve stability and restore aesthetic appearance through three-dimensional reduction and rigid fixation. The purpose of this study was to evaluate the stability and aesthetic appearance outcomes of one-point fixation using a three-dimensional photogrammetric analysis.

**Methods:**

From March 2014 to December 2014, 34 patients with ZMC fractures were treated by one-point fixation in the ZM buttress using unsintered hydroxyapatite (u-HA)/poly-L-lactide (PLLA) plates. Differences in soft tissue inter-malar height between the fractured side and unfractured sides were evaluated using photogrammetric analysis with a three-dimensional camera (Morphius®) at the preoperative and 1 week, 1 and 3 months after surgery. The differences in bony inter-malar height between the fractured and unfractured sides were evaluated using computer tomography at the pre-operative and 6 months after surgery. The paired *t*-test was used to compare differences in malar height.

**Results:**

Six months after surgery, 34 patients achieved satisfactory bony stability and symmetric malar appearances. Comparisons of differences in soft-tissue inter-malar height revealed statistically significant differences between the pre-operative period and 1 week and 1 month after surgery (*p* < .01). There was no statistically significant difference between 1 and 3 months after surgery. Comparison of differences in bony inter-malar height revealed a statistically significant difference between before and 6 months after surgery (*p* < .01).

**Conclusions:**

When we conducted a three-dimensional photogrammetric analysis, although it has restricted surgical indications, one-point fixation of the ZM buttress using an u-HA / PLLA plate yielded reliable, satisfactory, and safe clinical results in patients with ZMC fractures.

**Clinical question / level of evidence:**

Therapeutic, III.

## Introduction

Zygomaticomaxillary (ZM) complex (ZMC) fractures are common facial fractures [1, 2]. The ZMC is an important structure, serving as a major buttress of the middle third of the face. The ZMC also projects anterolaterally to form the malar eminence and establishes the midfacial width and contour of the orbital rim; thus, it plays an important role in terms of aesthetic appearance [3].

The goal of treating ZMC fractures is to achieve stability and restore aesthetic appearance through three-dimensional reduction and rigid fixation. After adequate reduction of the fracture has been achieved, it is important to maintain stability and rigid fixation to prevent functional impairment and aesthetic sequelae. Open reduction and internal fixation has been used as the standard method for treating ZMC fractures [4].

For the reduction of ZMC fractures, various surgical techniques including one-, two-, and three-point fixation have been introduced; these are based on the severity and extent of the fracture [5–9]. Some authors have argued that the three-point fixation method is essential to avoid facial asymmetry caused by delayed displacement [10]. However, some authors have also argued that the one-point fixation method provides sufficient stability of the ZMC fracture when the ZMC fracture is not comminuted [6–8, 11]. At this time, it remains unclear which treatment, one-point fixation, two-point fixation, or three-point fixation, is best [6, 10–12]. As interest in minimally invasive procedures and concerns about scarring have increased, many surgeons have come to prefer to treat ZMC fractures using one-point fixation. In particular, one-point fixation of the ZM buttress through a gingivobuccal incision has advantages in that it does not leave an external scar.

Unsintered hydroxyapatite (u-HA) particles and poly-L-lactic (PLLA) composites (OSTEOTRANS MX®; Takiron Co., Ltd., Japan) are new-generation absorbable devices. They are bioactive and totally resorbable osteosynthetic bone fixation devices consisting of a composite material with bioactive, bioresorbable u-HA and carbonated ion containing fine particles combined with PLLA [13, 14]. Some authors have reported that u-HA/PLLA composite may be appropriate for total replacement with bone [15].

We performed one-point fixation of a ZM buttress in the treatment of selected cases of ZMC fractures through a gingivobuccal incision using u-HA/PLLA composite devices. The purpose of this study was to evaluate the outcomes of our surgical procedure using three-dimensional photogrammetric analysis in terms of stability and aesthetic appearance.

## Methods

Of the patients who underwent open reduction and rigid fixation for ZMC fractures using u-HA/PLLA composites between March 2014 to December 2014, 34 who could be observed and followed for longer than 6 months were enrolled in this study. The inclusion criteria were type III, IV, and V ZMC fractures according to the Knight and North classification. Patients who had bilateral ZMC fractures, comminuted ZMC fractures, a previous history of craniofacial surgery, or a history of congenital facial asymmetry were excluded from this study. The study conformed to the Declaration of Helsinki, and written consent was obtained from each patient for both the surgery and the publication of the photographs of the results. The study was reviewed and approved by the Institutional Review Board for human subjects’ research (IRB no. SCHBC 2016–10–023-02).

### Surgical procedure

**Fig. 1:**
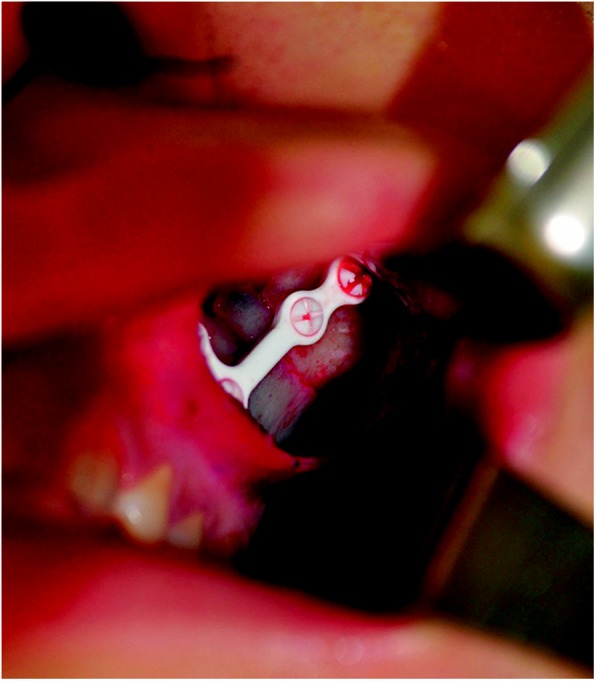
One-point fixation of the zygomaticomaxillary buttress region using unsintered hydroxyapatite particles/poly-L-lactide composites. The fracture site was fixed with a four-holed, 1.4-mm thick L-type plate and 8 mm screws

One surgeon treated all patients with ZMC fractures. Keen’s approach was used for reducing the fractures. All procedures were performed under general anesthesia with oroendotracheal intubation. A gingivobuccal incision was made 1 cm above the attached gingiva and then deepened through the buccinator straight to the anterior maxillary wall until the periosteum was identified. After the mucoperiosteal flap was elevated, fracture sites were identified. A Digman zygoma arch elevator could also have been placed accurately and applied to reduce bone fragments and correct the anatomical position. We performed one-point fixation at the ZM buttress region using u-HA/PLLA composites (Fig. 1). The buttress region was fixed with a four-holed L-type plate (1.4 mm thickness, 4.5 mm width, 22 × 10 mm) and 8 mm screws. After rigidity of the fixation was confirmed, the wound was copiously irrigated. Hemostasis was achieved, and watertight closure was obtained.

We recommended the maintenance of a soft diet for 1 month after surgery and a malar splint for the protection of the malar eminence for 1 week after surgery. All patients were administered prophylactic intravenous antibiotics (Oxacephem, 2000 mg/day) for 7 days after the operation.

### Clinical examination

**Fig. 2:**
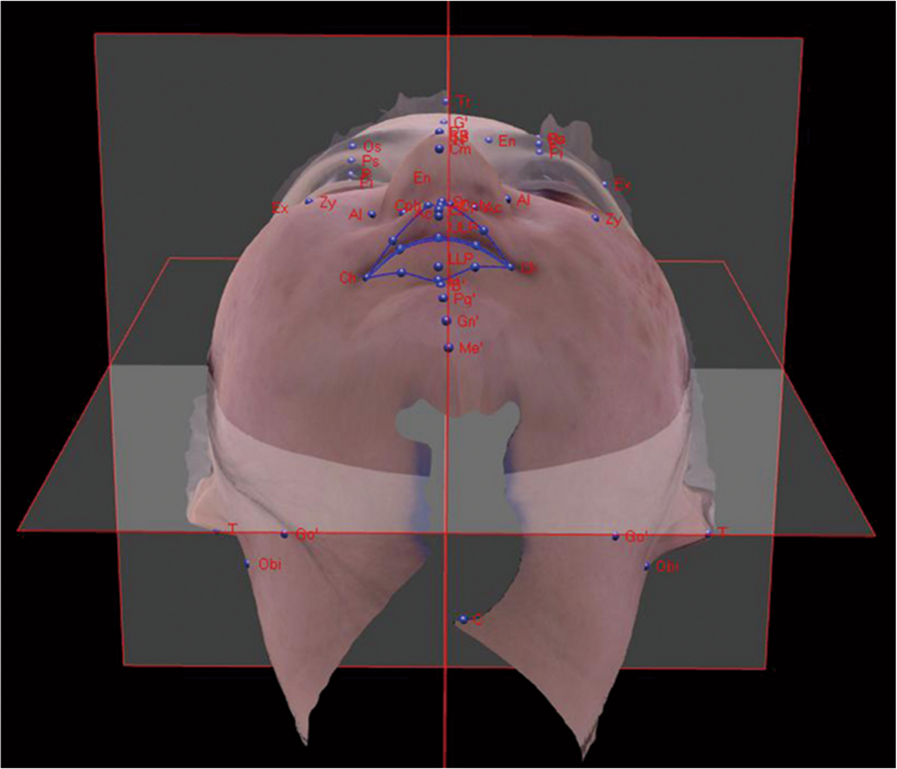
Differences in soft-tissue malar height between the fractured and unfractured sides were evaluated with photogrammetric analysis using a three-dimensional camera (Morpheus3D scanner)

All patients underwent a preoperative physical examination and were investigated using Water’s skull view and the zygomatic arch view. Differences in the soft-tissue malar height between the fractured and unfractured sides were evaluated with photogrammetric analysis using the three-dimensional (3D) camera (Morphius®, Morpheus3D Co., Ltd., Korea). For measurement of soft-tissue inter-malar height, we used an arbitrary reference line parallel to the intercanthal line. We turned up the 3D photography until the nasal tip was parallel to the eyebrow levels and measured the distances from the arbitrary reference line to the points at the height of the most prominent malar region (Fig. 2). Distance from the reference line to the points was defined as the soft tissue inter-malar heights and measured before and after surgery. Differences in the bony inter-malar heights between the fractured and unfractured sides were evaluated using 3D computed tomography (3D CT). The postoperative soft-tissue inter-malar height assessment was conducted with a 3D camera 1 week, 1 month, and 3 months after surgery. At 6 months after the operation, bony inter-malar height was evaluated using 3D CT. During the follow-up period after surgery, all patients underwent clinical and radiological assessment by independent observers.

### Statistical analysis

All variables are expressed as means ± standard deviation (SD) and obtained a 95% confidence interval. Statistical analyses were performed using SPSS software (version 18.0; SPSS Inc., Chicago, IL, USA). The paired *t*-test was used to compare differences in malar height, and *p*-values < .05 were considered significant.

## Results

Of the 34 patients, 26 were male and 8 were female. The mean age of the patients was 39.5 years (range 17–74 years). A total of 10 patients had an isolated ZMC fracture, and 24 had combined other facial bone fractures, such as orbital wall and nasal bone fractures (Table 1).

**Table 1:** Demographic characteristics of patients

Variable	Value
Sample size	34
Sex	
Male	26
Associated facial bone fracture	
None	10
Nasal bone fracture	8
Orbital wall fracture	19
Finger fracture	1
Age (year)	39.47 ± 3.04

**Fig. 3:**
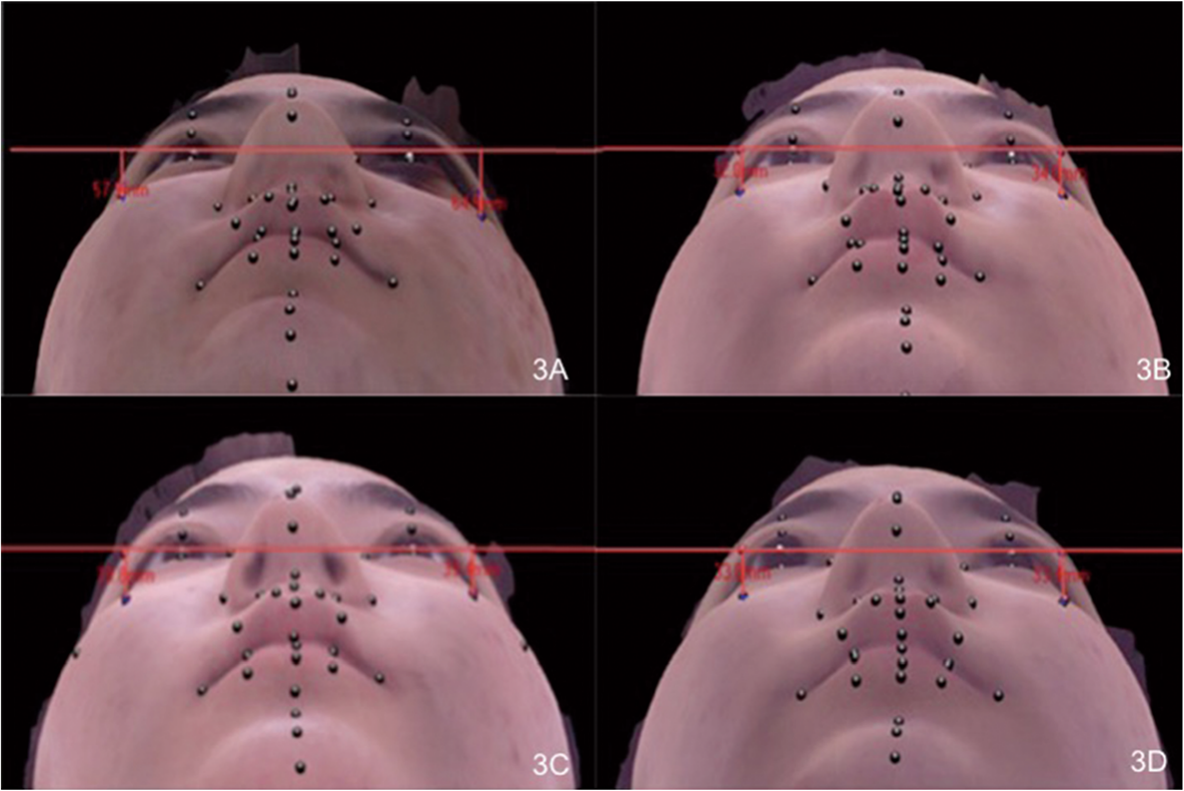
A 23-year-old female with a left zygomaticomaxillary complex fracture was treated with one-point fixation using unsintered hydroxyapatite particles/poly-L-lactide composites. The difference in soft-tissue malar height was evaluated with photogrammetric analysis using a three-dimensional camera (Morpheus3D scanner). **a** Preoperative photogrammetric analysis. **b** Photogrammetric analysis finding 1 week after surgery. **c** Photogrammetric analysis finding 1 month after surgery. **d** Photogrammetric analysis finding 3 months after surgery

**Fig. 4:**
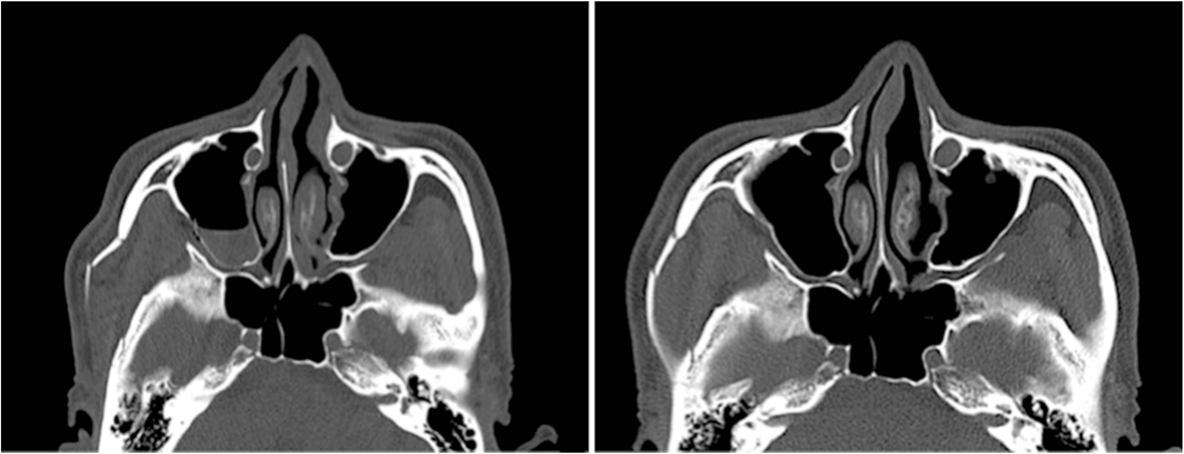
A 37-year-old male with a right zygomaticomaxillary complex fracture was treated with one-point fixation using unsintered hydroxyapatite particles/poly-L-lactide composites. Preoperative computed tomography (CT) image show lateral rotation of zygoma and depression of the zygomatic arch (left), and postoperative CT image show the reduction state 6 months after surgery (right)

The mean follow-up period was 7 months (range 6–9 months). During the hospitalization and follow-up periods, there were no major complications, such as wound infection, plate exposure or fracture, bony non-union, or infection-related foreign body reaction. All 34 patients achieved satisfactory symmetric soft tissue malar appearance 3 months after surgery (Fig. 3) and bony stability and symmetric bony malar appearance 6 months after surgery (Fig. 4).

**Fig. 5:**
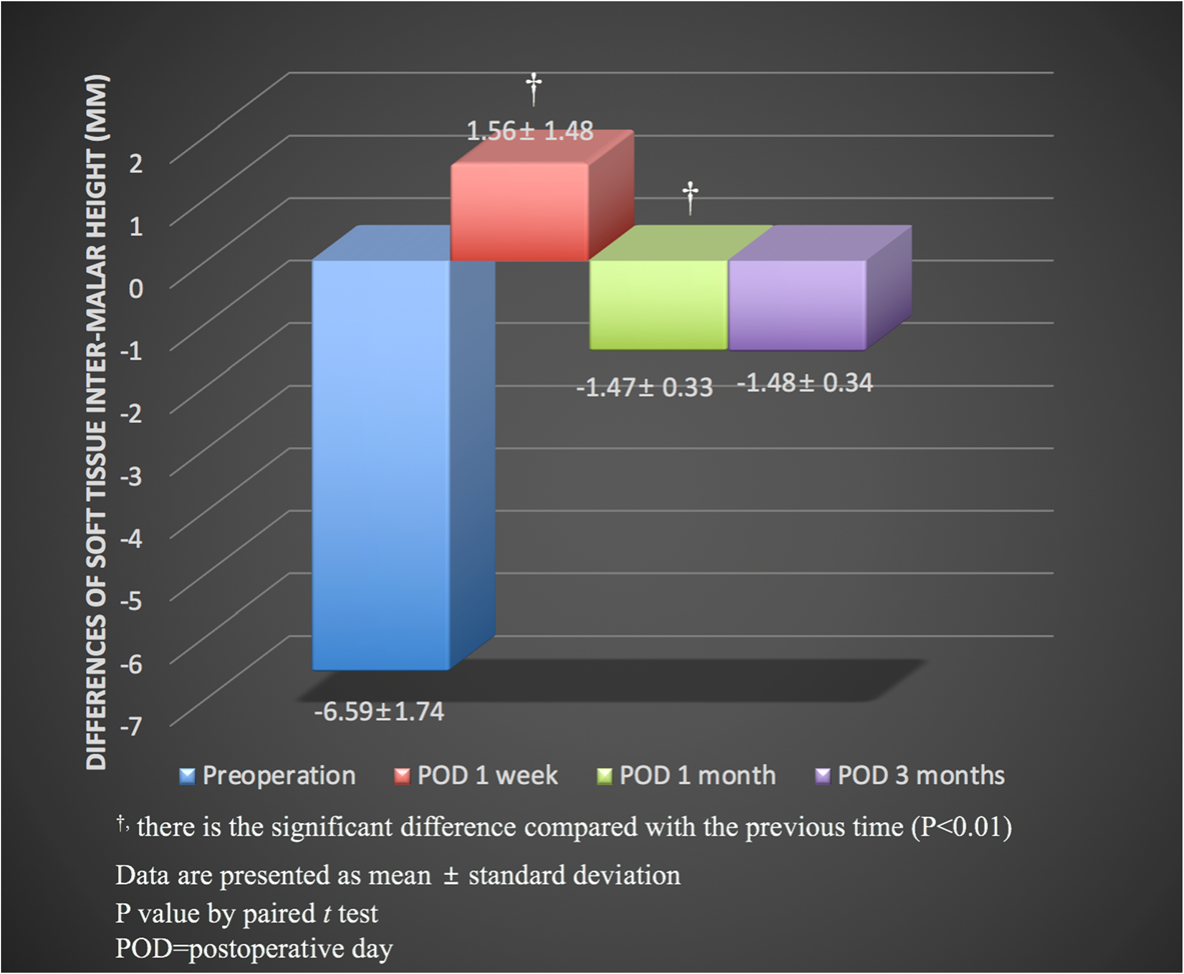
Preoperative differences in soft-tissue malar height between the fractured and unfractured sides were measured as − 6.59 ± 1.74 mm. It was 1.56 ± 1.48 mm at 1 week, − 1.47 ± 0.33 mm at 1 month, and − 1.48 ± 0.34 mm at 3 months after surgery. There were statistically significant differences between the preoperative and 1-week and 1-month postoperative figures (*p* < .01). There were no statistically significant differences between postoperative 1 month and postoperative 3 months (*p* = .08). ^†^There is the significant difference compared with the previous time (*p* < .01). *p*-value by paired *t*-test. POD = postoperative day

The average postoperative difference in soft-tissue inter-malar height between the fractured and unfractured sides was − 6.59 ± 1.74 mm (means ± standard deviation): it was 1.56 ± 1.48 mm at 1 week, − 1.47 ± 0.33 mm at 1 month, and − 1.48 ± 0.34 mm at 3 months (Fig. 5). The difference in the soft-tissue inter-malar heights before and 1 week after surgery was statistically significant (*p* < .01). The difference in the soft-tissue inter-malar heights 1 week and 1 month after surgery was statistically significant (*p* < .01). There was no statistically significant difference between these values at 1 and 3 months after surgery.

**Fig. 6:**
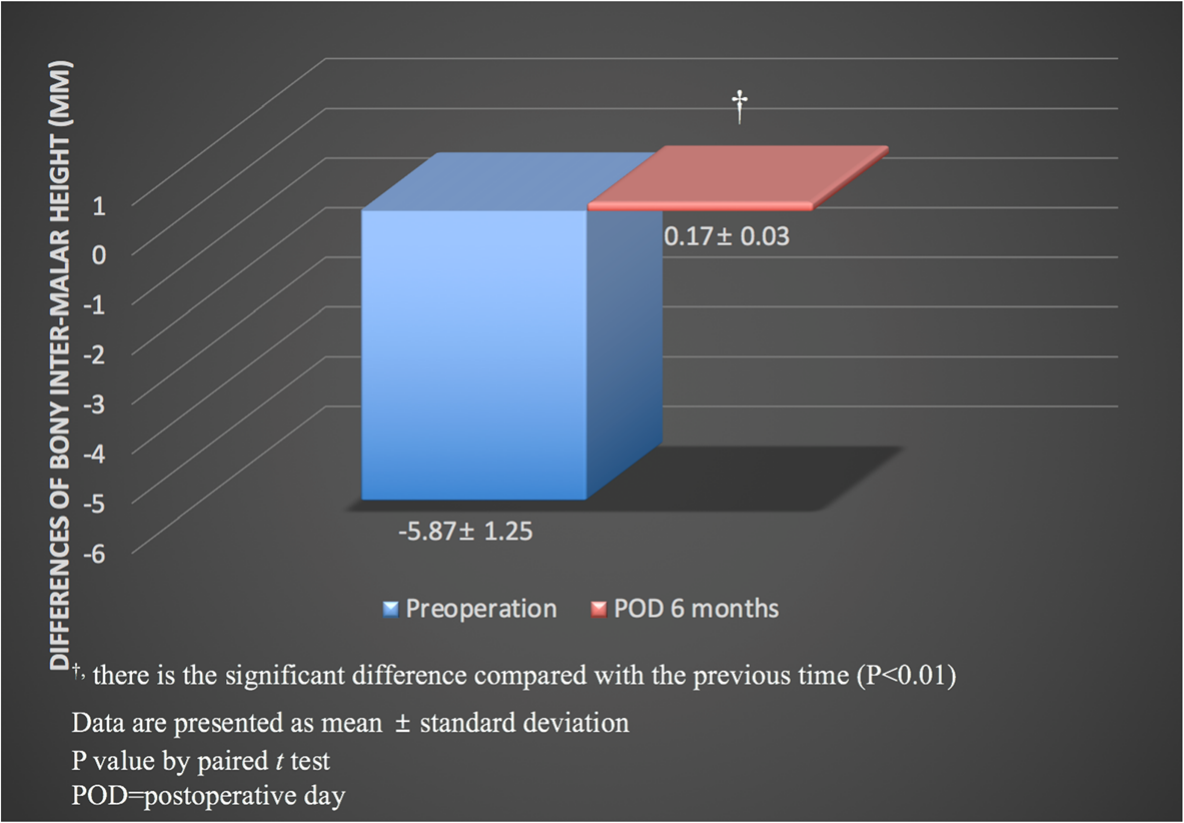
Differences in bony malar height were − 5.78 ± 1.25 mm before surgery and 0.17 ± 0.03 mm at 6 months after surgery, and there was a statistically significant difference between before and 6 months after surgery (*p* < .01). ^†^There is the significant difference compared with the previous time (*p* < .01). *p*-value by paired *t*-test. POD = postoperative day

The differences in bony inter-malar height were − 5.78 ± 1.25 mm before and 0.17 ± 0.03 mm 6 months after surgery, which reflect a significant difference (*p* < .01) (Fig. 6).

## Discussion

The ZMC is commonly injured because of its prominent location and contour. The ZMC has four suture lines and three principle buttresses that give the ZMC an intrinsic strength, and injury typically results in tetrapod or tripod fractures [16]. Functional impairment, such as restricted mouth opening, sensory deficits, enophthalmos, diplopia, or facial asymmetry can arise from inadequate reduction or secondary displacement following initial reduction as a result of masticatory forces [17–19]. Therefore, it is important to achieve stability and restore natural function for ZMC fracture patients.

To overcome physiological stresses and maintain stability at the fracture sites, three-point fixation may provide the best stability [5, 20]. However, previous studies found that two-point fixation [9, 21] and one-point fixation [6–8, 12, 22] are also sufficient to maintain stability and show good results in selected cases. Complications, such as infection, nerve injury, and incision scars, can be avoided by these less invasive procedures. Recently, patients have become increasingly concerned about whether their aesthetic appearance will be restored.

To maintain stability through one-point fixation, it is important that osteosynthetic devices have a high degree of strength. The ideal osteosynthetic device should not only maintain adequate strength throughout bone healing but also be safe for clinical use. Titanium materials have resistance to corrosion, excellent tensile strength, and good handling properties. Although they are commonly used as standard osteonsynthetic devices in the craniofacial region, titanium osteosynthetic devices remain at the fracture site, resulting in complications, such as palpability, pain, cold intolerance, hypersensitivity, and exposure [23–25]. There is no consensus regarding the need for routine removal of asymptomatic titanium materials, but symptomatic titanium materials should be removed when clinically indicated [26–28]. In some cases, loose titanium devices may migrate and become visible or palpable in facial areas where the dermis and subcutaneous tissues are relatively thin [26, 29]. Because of the limited bone growth, titanium osteosynthetic devices should be removed, especially in pediatric patients [30, 31]. These can also produce artifacts on computed tomography or magnetic resonance imaging [32–34].

Various absorbable osteosynthetic devices have been developed to address these problems of titanium materials, and they have been used in the cranofacial regions for trauma and reconstructive procedures. However, conventional absorbable osteosynthetic devices may have some limitations, including mechanical weakness. Several studies have shown that absorbable materials are mechanically insufficient for fracture stabilization, and this can result in impaired bone healing, especially at the infraorbital rim and the ZM buttress [35, 36]. To overcome this drawback, a new-generation bioresorbable osteosynthetic device has been developed. A reinforced composite material, which is composed of noncalcined, u-HA/PLLA and has bioresorbability and osteoconductivity, has shown enhanced strength with rigidity and the ability to remain strong during the bone healing period [18]. Its initial bending strength of up to 270 megapascals is stronger than that of cortical bone, and its initial bending modulus of 12 gigapascals is close to that of cortical bone [37]. The bioresorbable u-HA particles in the device provide osteoconductivity and further improvement in terms of biocompatibility and safety in vitro and in vivo [14, 38, 39]. Compared with studies using other absorbable materials, u-HA/PLLA composites show higher stability in segmental retention [40].

The hydroxyapatite that one of the main components of u-HA/PLLA materials is not a resorbable materials. However, u-HA particles are synthesized by hydrolysis of calcium hydrogenphosphate anhydrate (CaHPO4) in the presence of calcium carbonate (CaCO3) [13]. The average size range of u-HA particles (3–5 μm) is suitable for phagocytosis and incorporation into the PLLA matrix [15, 37]. Biodegradation of the u-HA/PLLA materials is carried out as follows: gradual loss of strength, resorption of PLLA, which has degraded to a low molecular weight, and release of unbound of HA debris and deposition of calcium phosphates [13]. Shikinami et al. [15] reported that the u-HA/PLLA material in the femoral bone of rabbit decreases gradually in sizes as low PLLA molecules and unbound u-HA particles are steadily released over 2.5 years after implantation. However, the postoperative complications related the u-HA/PLLA plate were reported. Landes et al. [38] reported that they experienced two patients had foreign body reactions, with local redness and swelling at 15 and 33 months after operation and their symptoms were resolved by plate removal and curettage. Hayashi et al. [13] reported that they experienced the bone excess on the frontal bone at 6 months after operation and the inflammatory granulation tissue on the frontozygomatic suture site at 2 years after operation. By referring to these articles, we only included patients with mild and moderate displacement who can be treated by one-point fixation in ZM buttress region to avoid complications related u-HA/PLLA plate in our study.

Comparisons of the soft-tissue inter-malar height of the fractured and unfractured sides revealed statistically significant differences between before surgery and 1 week and 1 month after surgery. The difference in soft-tissue inter-malar height 1 week and 1 month after surgery may have been caused not only by the minor decrease in bony malar height due to the masseter muscle action but also by the reduction in postoperative soft-tissue swelling. However, there was no statistically significant difference between 1 and 3 months after surgery. It means that the change of soft tissue inter-malar height mainly occurs within 1 month after surgery. The differences in bony inter-malar height were 0.17 ± 0.03 mm 6 months after surgery. This result suggested that u-HA/PLLA composites may provide a sufficient rigidity of the ZM buttress to maintain bony alignment when one-point fixation methods are used.

Our study has several limitations. First, we included only those ZMC fractures with mild and moderate displacement. ZMC fractures with mild or moderate displacement require less bony stability and can tolerate a weaker osteosynthetic device compared with a severely displaced ZMC fracture. Therefore, the u-HA/PLLA composite osteosynthetic device can be used to treat selected cases of ZMC fracture. Second, our study had a short-term follow-up period and relatively few patients. The effectiveness and complications of u-HA/PLLA composites for ZMC fractures cannot be fully supported based on results obtained during a 6 months postoperative follow-up period. Although further studies with long-term follow-up periods are required, our study suggest that ZMC fractures with mild and moderate displacement can be treated with one-point fixation of the ZM buttress using u-HA/PLLA composites. Third, we evaluated the postoperative results using 3D digital photogrammetric analysis, but it is possible that this approach introduced a bias related to indirect anthropometry owing to its reliance on 3D digital photogrammetric analysis.

Despite these limitations, and although it has restricted surgical indications, one-point-fixation of the ZM buttress using u-HA/PLLA can provide sufficient bony stability without external scars. We suggest that our surgical technique offers an alternative treatment for ZMC fractures with mild and moderate displacement.

## Conclusions

This clinical study suggests that one-point fixation of a ZM buttress using u-HA/PLLA composites provides reliable, satisfactory, and safe clinical results in patients with ZMC fractures with mild and moderate displacement.

## Data Availability

The datasets generated during and/or analysed during the current study are available from the corresponding author on reasonable request.

## Abbreviations

3D: three-dimensional
CaHPO4: Calcium hydrogenphosphate anhydrate
CT: Computed tomography
PLLA: Poly-L-lactide
u-HA: unsintered hydroxyapatite
ZM: Zygomaticomaxillary
ZMC: Zygomaticomaxillary complex

## References

[1] Ellis E, Kittidumkerng W (1996). Analysis of treatment for isolated zygomaticomaxillary complex fractures. J Oral Maxillofac Surg.

[2] Hwang K, Kim DH (2011). Analysis of zygomatic fractures. J Craniofac Surg..

[3] Ribeiro MC, Regalo SC, Pepato AO, Siéssere S, de Souza LG, Sverzut CE (2011). Bite force, electromyography, and mandible mobility during the 6-month period after surgical treatment for isolated fractures of the zygomatico-orbital complex. Oral Surg Oral Med Oral Pathol Oral Radiol Endod.

[4] Yonehara Y, Hirabayashi S, Tachi M, Ishii H (2005). Treatment of zygomatic fractures without inferior orbital rim fixation. J Craniofac Surg..

[5] Davidson J, Nickerson D, Nickerson B (1990). Zygomatic fractures: comparison of methods of internal fixation. Plast Reconstr Surg.

[6] Kim JH, Lee JH, Hong SM, Park CH (2012). The effectiveness of 1-point fixation for zygomaticomaxillary complex fractures. Arch Otolaryngol Head Neck Surg.

[7] Hwang K (2010). One-point fixation of tripod fractures of zygoma through a lateral brow incision. J Craniofac Surg..

[8] Kim ST, Go DH, Jung JH, Cha HE, Woo JH, Kang IG (2011). Comparison of 1-point fixation with 2-point fixation in treating tripod fractures of the zygoma. J Oral Maxillofac Surg.

[9] Rana M, Warraich R, Tahir S, Iqbal A, von See C, Eckardt AM (2012). Surgical treatment of zygomatic bone fracture using two points fixation versus three point fixation--a randomised prospective clinical trial. Trials..

[10] Holmes KD, Matthews BL (1989). Three-point alignment of zygoma fractures with miniplate fixation. Arch Otolaryngol Head Neck Surg..

[11] Schilli W, Ewers R, Niederdellmann H (1981). Bone fixation with screws and plates in the maxillo-facial region. Int J Oral Surg.

[12] Fujioka M, Yamanoto T, Miyazato O, Nishimura G (2002). Stability of one-plate fixation for zygomatic bone fracture. Plast Reconstr Surg.

[13] Hayashi M, Muramatsu H, Sato M, Tomizuka Y, Inoue M, Yoshimoto S (2013). Surgical treatment of facial fracture by using unsintered hydroxyapatite particles/poly l-lactide composite device (OSTEOTRANS MX((R))): a clinical study on 17 cases. J Craniomaxillofac Surg.

[14] Landes CA, Ballon A, Tran A, Ghanaati S, Sader R (2014). Segmental stability in orthognathic surgery: hydroxyapatite/poly-l-lactide osteoconductive composite versus titanium miniplate osteosyntheses. J Craniomaxillofac Surg.

[15] Shikinami Y, Matsusue Y, Nakamura T (2005). The complete process of bioresorption and bone replacement using devices made of forged composites of raw hydroxyapatite particles/poly l-lactide (F-u-HA/PLLA). Biomaterials..

[16] Rohrich RJ, Watumull D (1995). Comparison of rigid plate versus wire fixation in the management of zygoma fractures: a long-term follow-up clinical study. Plast Reconstr Surg.

[17] Bogusiak K, Arkuszewski P (2010). Characteristics and epidemiology of zygomaticomaxillary complex fractures. J Craniofac Surg.

[18] Manson PN, Crawley WA, Yaremchuk MJ, Rochman GM, Hoopes JE, French JH (1985). Midface fractures: advantages of immediate extended open reduction and bone grafting. Plast Reconstr Surg.

[19] Zingg M, Laedrach K, Chen J, Chowdhury K, Vuillemin T, Sutter F (1992). Classification and treatment of zygomatic fractures: a review of 1,025 cases. J Oral Maxillofac Surg.

[20] Rohrich RJ, Hollier LH, Watumull D (1992). Optimizing the management of orbitozygomatic fractures. Clin Plast Surg.

[21] Haidar Z (1978). Fractures of the zygomatic complex in the south-east region of Scotland. Br J Oral Surg.

[22] Mohammadinezhad C (2009). Evaluation of a single miniplate use in treatment of zygomatic bone fracture. J Craniofac Surg..

[23] Alpert B, Seligson D (1996). Removal of asymptomatic bone plates used for orthognathic surgery and facial fractures. J Oral Maxillofac Surg.

[24] Meningaud JP, Poupon J, Bertrand JC, Chenevier C, Galliot-Guilley M, Guilbert F (2001). Dynamic study about metal release from titanium miniplates in maxillofacial surgery. Int J Oral Maxillofac Surg.

[25] Nagase DY, Courtemanche DJ, Peters DA (2005). Plate removal in traumatic facial fractures: 13-year practice review. Ann Plast Surg.

[26] Bhatt V, Chhabra P, Dover MS (2005). Removal of miniplates in maxillofacial surgery: a follow-up study. J Oral Maxillofac Surg.

[27] Matthew IR, Frame JW (1999). Policy of consultant oral and maxillofacial surgeons towards removal of miniplate components after jaw fracture fixation: pilot study. Br J Oral Maxillofac Surg.

[28] Verweij JP, Houppermans PN, Mensink G, van Merkesteyn JP (2014). Removal of bicortical screws and other osteosynthesis material that caused symptoms after bilateral sagittal split osteotomy: a retrospective study of 251 patients, and review of published papers. Br J Oral Maxillofac Surg.

[29] Chaushu G, Manor Y, Shoshani Y, Taicher S (2000). Risk factors contributing to symptomatic plate removal in maxillofacial trauma patients. Plast Reconstr Surg.

[30] Eppley BL, Platis JM, Sadove AM (1993). Experimental effects of bone plating in infancy on craniomaxillofacial skeletal growth. Cleft Palate Craniofac J.

[31] Iatrou I, Theologie-Lygidakis N, Tzerbos F (2010). Surgical protocols and outcome for the treatment of maxillofacial fractures in children: 9 years' experience. J Craniomaxillofac Surg.

[32] Fiala TG, Paige KT, Davis TL, Campbell TA, Rosen BR, Yaremchuk MJ (1994). Comparison of artifact from craniomaxillofacial internal fixation devices: magnetic resonance imaging. Plast Reconstr Surg.

[33] Peltoniemi HH, Ahovuo J, Tulamo RM, Törmälä P, Waris T (1997). Biodegradable and titanium plating in experimental craniotomies: a radiographic follow-up study. J Craniofac Surg..

[34] Tschakaloff A, Losken HW, Lalikos J, Link J, Mooney MP, von Oepen R (1993). Experimental studies of DL-polylactic acid biodegradable plates and screws in rabbits: computed tomography and molecular weight loss. J Craniofac Surg..

[35] Pohjonen T, Helevirta P, Törmälä P, Koskikare K, Pätiälä H, Rokkanen P (1997). Strength retention of self-reinforced poly-L-lactide screws. A comparison of compression moulded and machine cut screws. J Mater Sci Mater Med.

[36] Wittwer G, Adeyemo WL, Voracek M, Turhani D, Ewers R, Watzinger F (2005). An evaluation of the clinical application of three different biodegradable osteosynthesis materials for the fixation of zygomatic fractures. Oral Surg Oral Med Oral Pathol Oral Radiol Endod..

[37] Shikinami Y, Okuno M (1999). Bioresorbable devices made of forged composites of hydroxyapatite (HA) particles and poly-L-lactide (PLLA): part I. Basic characteristics Biomaterials.

[38] Landes C, Ballon A, Ghanaati S, Tran A, Sader R (2014). Treatment of malar and midfacial fractures with osteoconductive forged unsintered hydroxyapatite and poly-L-lactide composite internal fixation devices. J Oral Maxillofac Surg.

[39] Ishii S, Tamura J, Furukawa T, Nakamura T, Matsusue Y, Shikinami Y (2003). Long-term study of high-strength hydroxyapatite/poly(L-lactide) composite rods for the internal fixation of bone fractures: a 2-4-year follow-up study in rabbits. J Biomed Mater Res B Appl Biomater.

[40] Buijs GJ, van Bakelen NB, Jansma J, de Visscher JG, Hoppenreijs TJ, Bergsma JE (2012). A randomized clinical trial of biodegradable and titanium fixation systems in maxillofacial surgery. J Dent Res.

